# Supplementary material for: Physiotherapists clinical reasoning to prescribe exercise for patients with chronic pain: A qualitative study research protocol
Source: PLoS One. 2023 Dec 1;18(12):e0295382. doi: 10.1371/journal.pone.0295382 (PMC10691676; doi:10.1371/journal.pone.0295382)
Supplement: S1 File — (DOCX) [file pone.0295382.s001.docx]

| Part of interview | Themes to cover | Prompts/specific questions |
| --- | --- | --- |
| Arrival and introduction | -Welcome  -Overview of research  -Overview of interview  -Confirmation of consent to proceed |  |
| Opening questions | -What do you feel is the most important aspect of managing patients with persistent/chronic pain?  -What is your approach to managing persistent pain?  -Where does exercise and physical activity (PA) fit into how you manage patients?  -Do you make distinction between PA and exercise?  -If so, how does it help, what influences you to look more for PA versus exercise prescription  -If not, why is this? | -Is it due to limited evidence in terms of exercise specifics/dose, or unaware of difference, or don’t feel it impacts on reasoning |
| Prescription | -When you prescribe exercise/PA- broadly, what are the key influences?  - Have the guidelines impacted on your reasoning in terms of exercise/PA prescription?  -Are you aware of the NICE guidelines for exercise/PA in persistent pain, if so, what are your views of them?  -How do you choose the exercise modality? for example resistance or aerobic exercise?  What are the key influences on this, and how specific do you prescribe PA/Exercise? -How much detail do you consider, when prescribing exercise?  -What parameters do you use to guide your prescription?  - Do you have very specific goals with the prescription? If so, how are these developed and how do they influence your clinically reasoning?  -If the reduction of pain is a goal, how do you prescribe the exercise/PA to achieve this? What is the rationale and underpinning reasoning for how the prescription will reduce pain?  -How do you measure or attain a baseline for exercise/PA prescription? What are the key aspects that inform your clinical reasoning?  -What are the key variables that you manipulate to help attain the goals?  -Progression, regression, measurement- what influences your decisions in terms of how progression (rate/magnitude) is applied. Are there key markers/milestones used and considered? Is this the same for how you regress exercise or manage a flare up of symptoms? How do you clinically reason in relation to these factors?  -Do you consider pacing with your exercise prescription, if so, how do use this and how much does this influence your clinical reasoning in terms of your prescription? Is pain response key, and how does it influence on your decision for progression/regression?  -Pain: if this is a key goal, how do you dose to reduce/manage this?  -what are the key influences on this, and approaches to doing so.  -Are you looking for a specific “dose”, and does this depend on a baseline measurement(s)? | -How detailed will you prescribe, and is this based on non-pain focused goals (i.e. strength/power/muscular endurance for example) or is pain, or function, or another goal more important?  -If there is a very specific goal, is this the driver behind how you prescribe the exercise? So, for example, if you wanted to increase strength, does this drive specifically how you prescribe the exercise?  -What are the mechanisms by which the exercise/PA will reduce pain, why do you specifically prescribe exercise/PA in the manner that you do?  -What is your approach to gaining a baseline, what are the influences on how you assess this, if you do so?  -Examples of variables could be sets, reps, %RM for example. |
| Patient presentation and influence | -As a patient, how much influence do they have on the PA/exercise they are prescribed?  -Are there any patient specific influences (age, sex, socio-economic background, pain condition, values and goals) and patient preferences in addition/aligned to goals.  -Are there any specific psychological or environmental factors that influence your exercise prescription?  -How does the pain condition specifically (FMS, CLBP, OA etc.) influence your reasoning- does the pain mechanism provide specific influence? |  |
| Personal, environmental and professional influences | -Beyond what has been discussed, what other personal or professional influences are there on your reasoning?  -Are there any environmental factors that influence your reasoning?  -How do the influences interact? Is there a hierarchy? Which provide the most influence? Is there an overriding area? | -Influence of training courses, departmental IST’s  -Influence of the working environment, colleagues and training.  -Influence by social media feeds (Twitter, Facebook etc,)  -Influenced by specific evidence-based pain literature (books, articles)  -Influenced by guru driven ideals  -Influenced by historical ideals (3x10- no one can specify source but passed down over generations)- if for example, 3x10, but with what resistance, what is the aim when using 3x10- is it progressive  -Influenced by environmental factors (available resources, gym etc.)  -Influenced by local service agreements  -Influenced by therapist experience (age, years working)  -Therapist personality influences and biases (sporty vs sedentary physio, competitive vs fun)  -Influenced by non-pain specific literature (Gabbet ACWR, sport science) |
