# Supplementary material for: Physiotherapists clinical reasoning to prescribe exercise for patients with chronic pain: A qualitative study research protocol
Source: PLoS One. 2023 Dec 1;18(12):e0295382. doi: 10.1371/journal.pone.0295382 (PMC10691676; doi:10.1371/journal.pone.0295382)
Supplement: Attachment — Submitted filename: Response to reviewers.docx [file pone.0295382.s002.docx]

Dear reviewers,

Thank you for taking the time to review our manuscript, and for providing clear and detailed feedback to help improve the submission. Below, we have provided a table with reviewer comments in one column and responses in another, to help provide a clear audit of the changes made. We have responded to each reviewers points separately, though some points made by the reviewers highlight the same issue (such as terminology relating to exercise and physical activity).

| Reviewer comments | Response(s) |
| --- | --- |
| Reviewer 1 |  |
| INTRODUCTION  • You are using exercise and physical activity interchangeably. I suggest you stick to exercise and not physical activity since physical activity cannot be prescribed (line 150, 155). It is exercise that can be prescribed. | Agreed, exercise will be utilised throughout. Reviewer 2 requested that exercise and physical activity should be defined, which has been added (lines 96-104) and is followed by why exercise will be used throughout the manuscript. |
| METHODS A lot of information has been mixed up in the methods section. Authors are providing information that is not necessary for a publication. For example, some details (lines 215 to 252) can be summarized in a single sentence and then just referenced. Authors are writing the methodology section as if this is a thesis, yet this is a manuscript for publication. I suggest you cut down any unnecessary details in the methods section and write the methodology section concisely with the following subheadings:  • Study design and type • Study place • Study population • Sample size/ sampling • Data collection • Data management and analysis • Ethical considerations • Dissemination of results | An effort was made to be more succinct in reporting of the methods in lines 207-227. However, reviewer two commented that a definition was required relating to information power/saturation, therefore this has remained in the script.  We have amended the script and used the titles and order of titles as suggested. However, we have combined study place/population as a sub heading, as each section in isolation would have limited detail.  We have added a sub heading for ethical considerations (lines 263-268) and dissemination of results respectively (270-273) |
| Some further comments in the methods section are below:  Study design • Information under study is largely describing the data collection process and the tools used for data collection. Authors need to indicate what study design will be used as this has not been described under the study design section. • Some information in lines 181 to 187 is misplaced. This information should come the ethical consideration. In addition, this section (ethical considerations) is missing and needs to be included. | We have clarified that this will be a qualitative study, following a generic qualitative approach as described by Kahlke (2014) on lines 172-175  Ethics information has been moved to the “ethical considerations” section (lines 263-268) |
| Study population • Information on sample size has been misplaced (lines 253 to 263) under data collection. This information should be under a sub heading sample size which follows the study population sub section. • Under sample size, authors should be specific on how participants will be sampled. What sampling frame will be used? | We have moved the information on sample size to the appropriate sub heading of sample size (lines 206-227)  As described in lines 190, a purposive sample will be used from the recruitment method outlined in lines 196-199. We felt that this approach was most appropriate, due to the nature of the physiotherapists linked to these associations. |
| Data analysis • Provide information on how data trustworthiness will be ensured. | This has been added in lines 259-261 |
| DISCUSSION  Study limitations • Since this is a protocol, indicate how you intend to mitigate the limitations that you foresee. | We have highlighted that the main limitation is the transferability of the study, due to the specific type of clinicians to be recruited, and highlighted how this could be mitigated in lines 313-314 |
|  |  |
| Reviewer 2 |  |
| Background – uses NICE abbreviation, suggest no abbreviations on an abstract, spell out in full for international readers. | Agreed, this has been amended accordingly. |
| Can we avoid using the word patients as many service users do not view themselves as patients, particularly in the outpatient setting | We have elected to keep the word patient, this is to keep aligned with the terminology used by National Institute for Health and Care Excellence (NICE), and the International Association for the Study of Pain (IASP). |
| Methods – again using UK abbreviation Write out in full | Agreed, this has been amended accordingly. |
| Ethics statement – needs more detail. Please explain why NHS IRAS/HRA ethics will not be required. What do you mean consent will be gained in the written format. Please give further details. Has ethics already been obtained? | Yes, ethics has been obtained. This was stated in lines 187-188 (in the original submission), detailing the ethics committee, reference number, and the date ethics was obtained. With the amendments to the manuscript, this is now on lines 263-268  Consent will be electronic, via email. This has been clarified in lines 200-204  As recruitment is not taking place directly within the NHS, but via social media, the Physiotherapy pain association and the Physiotherapy research society and can include participants that are not working in the NHS, NHS IRAS/HRA ethics is not required. We have highlighted in the manuscript that recruitment will not be directly within the NHS (lines 196-199) |
| Data section (page 5). I am confused as you state that no data sets were produced or analysed as part of the study but this study is generating a new data set (qualitative data from interviews). Please clarify and amend | The data collection has not started. For clarity, we have amended the date of the recruitment to summer 2023 (line 190). We did not want to/have not started data collection, as we wanted this protocol paper to be accepted first as per the PLOS one guidelines. |
| Full manuscript Background - You state that chronic pain and persistent pain are more or less the same. Why have you chosen to call it persistent pain throughout the manuscript as this could be confusing for your reader? | We have amended this to chronic throughout the script, which includes the title |
| - Chronic/persistent pain is complex. Are you focusing on clinical reason in certain conditions/populations (i.e MSK related, organ related, multi-morbidity, fibromyalgia)? Clarifying this would help focus the mind of your readers. | This has been clarified in the participants section (lines 190-195) and uses IASP references to support |
| - Please define exercise and physical activity (as they are two distinct, though related terms) | We have defined both terms (lines 96-104), as physical activity is discussed in the NICE guidelines, but also highlight why exercise will be used throughout the manuscript |
| - Although you have focused on physiotherapists as the main prescribers of exercise, I think you could expand the final paragraph of this section (page 14) to consider how other professionals could learn from these findings. For example exercise physiologists etc | We have added to the final paragraph, but opened this up to health care professionals (line 298 |
| Method - You state that the interview guide has been developing using literature. Can you elaborate? Have you completed a systematic/mapping review to identify literature? How can you be sure there isn’t any available evidence? - Please confirm if consent will be written or electronic | A systematic review/mapping review was not undertaken. However, the scope of the literature, as far as the research team was aware was used, due to the limited exploration of this subject. We had highlighted in the script that the scope of the literature as far as the research team was aware was utilised (lines 175-177)  Consent is electronic (lines 200-204) |
| - Robust qualitative research includes reflexive diaries. I note you have said that you will keep field notes but reflexive diaries are also needed and should be incorporated into the analytical framework | This has been added in lines 260-261 |
| - I am unclear why you need two people to complete a semi-structured interview. Why would JN observe and make notes? This is unsual for this method so please explain the rationale. The bias of two interviewers to one participant could potentially introduce bias into the method through social power. This needs to be explained further | We have removed JN from attending the interviews. |
| - If you are using data saturation to determine your sample size please define your working definition of saturation. | We are not using data saturation, but information power, which is covered in lines 211-216 |
| - There is limited detail on the analysis section. I note that you are suing the framework method which has seven stages of analysis (linked to the Gale reference). The authors have not followed this nor provided enough detail on their analytical method. For this to be published the seven stages need to be outlined. | We have added the seven stages of framework analysis to this section (lines 251-253) but have not discussed each, as the study has not yet been conducted. |
|  |  |
