# Supplementary material for: Physiotherapists clinical reasoning to prescribe exercise for patients with chronic pain: A qualitative study research protocol
Source: PLoS One. 2023 Dec 1;18(12):e0295382. doi: 10.1371/journal.pone.0295382 (PMC10691676; doi:10.1371/journal.pone.0295382)
Supplement: Attachment — Submitted filename: PONE-D-23-00756R1 Reviewer 1.docx [file pone.0295382.s003.docx]

I would like to congratulate the work done previously by the authors and very concise help and valuable help by the reviewers, in the presentation of the manuscript.

I have 2 minor additions which I suggest be added to the manuscript.

Line 95: What is your understanding/theoretical stand point in relation to complex pain, you state which approach is needed but not how you define and understand complex pain. This could be clarified.

95: “Management of chronic pain is complex, requiring a person-centred assessment [1]and multimodal approach [3].”

Clinical reasoning is a key component

Line 146-147 you define clinical reasoning for physiotherapists as a “context dependent way of thinking and decision making in professional practice to guide practice actions” [18]

Even though you describe an inductive approach in your framework analysis it seems unrealistic and maybe critical, that you do not have an informed starting point by having a model/theoretical understanding of clinical reasoning. This should be elaborated and clarified in the analysis section in Methods as this will influence the future analysis.
