# Supplementary material for: Physiotherapists clinical reasoning to prescribe exercise for patients with chronic pain: A qualitative study research protocol
Source: PLoS One. 2023 Dec 1;18(12):e0295382. doi: 10.1371/journal.pone.0295382 (PMC10691676; doi:10.1371/journal.pone.0295382)
Supplement: Attachment — Submitted filename: RESPON~1.DOC [file pone.0295382.s004.DOC]

Dear reviewers,

Thank you for taking the time to review our manuscript, and for providing clear and detailed feedback to help improve the submission. Below, we have provided a table with reviewer comments in one column and responses in another, to help provide a clear audit of the changes made.

| Reviewer comments | Response(s) |
| --- | --- |
| Line 95: What is your understanding/theoretical stand point in relation to complex pain, you state which approach is needed but not how you define and understand complex pain. This could be clarified.  95: “Management of chronic pain is complex, requiring a person-centred assessment [1]and multimodal approach [3].” | Within the script, we have used the term chronic pain, supported by the NICE guidelines definition. We have not used the term complex pain, as we are stating that the management of chronic pain is in itself complex (and requires person-centred assessment and multimodal approach). The focus of the study is clinical reasoning within chronic pain, as opposed to within complex pain, which may be a separate entity.  As we are not focusing on complex pain (which we are unaware if an accepted definition exists, or is an accepted term), we feel that discussing complex pain, or introducing this term to the paper adds unnecessary deviation from the focus of the study, namely chronic pain. |
| Clinical reasoning is a key component  Line 146-147 you define clinical reasoning for physiotherapists as a “context dependent way of thinking and decision making in professional practice to guide practice actions” [18]  Even though you describe an inductive approach in your framework analysis it seems unrealistic and maybe critical, that you do not have an informed starting point by having a model/theoretical understanding of clinical reasoning. This should be elaborated and clarified in the analysis section in Methods as this will influence the future analysis. | Within the analysis section of the methods section (line 256), we have highlighted that relevant clinical reasoning frameworks will inform analysis where appropriate, using an inductive approach to allow themes to emerge.  As different clinicians may utilise different clinical reasoning models (such as hypothetico-deductive, pattern recognition, biopsychosocial etc), we do not want to have a specific reasoning model as a starting point of analysis. |
