# Supplementary material for: Physiotherapists clinical reasoning to prescribe exercise for patients with chronic pain: A qualitative study research protocol
Source: PLoS One. 2023 Dec 1;18(12):e0295382. doi: 10.1371/journal.pone.0295382 (PMC10691676; doi:10.1371/journal.pone.0295382)
Supplement: Attachment — Submitted filename: Response to reviewers.docx [file pone.0295382.s005.docx]

Dear reviewers,

Thank you for taking the time to review our manuscript, and for providing clear and detailed feedback to help improve the submission. Below, we have provided a table with reviewer comments in one column and responses in another, to help provide a clear audit of the changes made.

| Reviewer comments | Response(s) |
| --- | --- |
| Kindly modify your abstract as previous into a structured abstract as follows with the following sub-headings, as recommended by the peer-reviewer: 1. Background (here include the purpose /aim of the study with a brief background). 2. Methods (summary of methods) 3. Results (summary of results/expected outcomes) 4. Conclusion | 1) We have added the aim of the study into the background. 2) The method has remained the same 3) As this is a protocol, we do not have any results. Therefore, we have included here what the outcome will be with the results. 4) We have removed the subtitle discussion and replaced with conclusion, and provided a conclusion. |
|  |  |
